# The Role of Gravity in the Evolution of the Concentration Field in the Electrochemical Membrane Cell

**DOI:** 10.3390/e22060680

**Published:** 2020-06-18

**Authors:** Kornelia M. Batko, Andrzej Ślęzak, Wioletta M. Bajdur

**Affiliations:** 1 Department of Business Informatics, University of Economics, 40287 Katowice, Poland; 2 Department of Innovation and Safety Management Systems, Technical University of Czestochowa, 42200 Czestochowa, Poland; aslezak52@gmail.com

**Keywords:** membrane transport, single-membrane system, Kedem–Katchalsky equations, concentration polarization, osmosis, natural convection

## Abstract

The subject of the study was the osmotic volume transport of aqueous CuSO_4_ and/or ethanol solutions through a selective cellulose acetate membrane (Nephrophan). The effect of concentration of solution components, concentration polarization of solutions and configuration of the membrane system on the value of the volume osmotic flux ($J_{vi}^{r})$ in a single-membrane system in which the polymer membrane located in the horizontal plane was examined. The investigations were carried out under mechanical stirring conditions of the solutions and after it was turned off. Based on the obtained measurement results $J_{vi}^{r}$, the effects of concentration polarization, convection polarization, asymmetry and amplification of the volume osmotic flux and the thickness of the concentration boundary layers were calculated. Osmotic entropy production was also calculated for solution homogeneity and concentration polarization conditions. Using the thickness of the concentration boundary layers, critical values of the Rayleigh concentration number ($R_{C}^{r}$), i.e., the switch, were estimated between two states: convective (with higher $J_{vi}^{r}$) and non-convective (with lower $J_{vi}^{r}$). The operation of this switch indicates the regulatory role of earthly gravity in relation to membrane transport.

## 1. Introduction

The membrane is a selective barrier separating the interior of the cell from its surroundings and plays a key role in the biological cell [1]. Attempts have been made to apply some features of cell membranes in membrane technologies used in various fields of science, technology and medicine as well as in various industries for a long time. Therefore, studies on membrane transport processes are carried out in order to learn, among others, mechanisms of transport across cell membranes or the development of membrane technologies and techniques useful in medicine (hemodializer) and industrial technologies (bioreactors, biorefineries, modules for food processing and water treatment, wastewater treatment, etc.) [2]. Polymers constitute the majority of film-forming materials: polymers highly stable (e.g., polybenzimidazole, polyamide, polytriazole, cellulose acetate, cellulose triacetate, etc.) and biodegradable polymers (e.g., poly/lactic acid, cellulose, bacterial cellulose, chitozan, etc.) [3]. They provide membrane materials for osmotic-based membrane system [4,5].

The membrane diffusion processes occurring spontaneously in real conditions are accompanied by the phenomenon of concentration polarization [6,7,8,9]. It consists in changing the concentration field or density of solutions in the areas on both sides of the membrane caused by the creation of concentration boundary layers. These layers significantly reduce membrane transport, which leads to a reduction in the efficiency of membrane processes in industrial technologies [2]. In biological systems as well as microchip systems of artificial membranes, concentration creation can have positive impact due to the spontaneous regulatory properties of the value of flux through the membrane, which in turn translates into slowing the source of entropy, and thus, slowing down the aging of the system [10]. *S*-entropy is the only general physical quantity that indicates irreversible and one-way flow of processes, including biological processes [11]. This means that entropy is produced in any non-equilibrium thermodynamic system, including membrane systems. Local entropy production is the sum of four contributions: thermal, diffusion, viscous and chemical [12]. Under isothermal, non-viscous conditions and without chemical reactions, the diffusion contribution plays a major role. It also applies to membrane transport processes. 

All “earthly phenomena” occur in the resultant gravitational field, whose main source is the Earth, the Moon and the Sun. The research into the impact of gravity on the concentration (density) field, generated in the environment of the separation membrane of non-mechanically mixed solutions, began in the 1970s. In 1972, the pioneering paper of S. Przestalski and M. Kargol about the discovery of the phenomenon of graviosmosis was published [8]. These studies were undertaken and continued by researchers directly or indirectly associated with the scientists. So far, several hundred papers on this issue have been published [7,13,14,15,16,17,18,19,20].

In previous papers, the results of experimental studies on the volume osmotic flux ($J_{vi}^{r}$, *r* = *α*, *β*, *i* = 1, 2) and solute flux ($J_{i}^{r}$, *r* = *α*, *β*, *i* = 1, 2) were presented. The solutions separated by the membrane contained aqueous solutions of glucose and/or ethanol [15,16], potassium chloride and/or ammonia [19]. The first of these substances causes an increase in and the second decreases the density of solutions. The characteristics of $J_{vi}^{r}$ = *f*(Δ*C_i_*, *r* = *α*, *β*, *i* = 1, 2) and $J_{i}^{r}$ = *f*(Δ*C_i_*, *r* = *α*, *β*, *i* = 1, 2) presented in these papers are non-linear and show typical transitions from convective to non-convective state and inversely. However, for the same membrane, they differ in terms of details that are related to the physico-chemical properties of the solutions. These papers also showed that the value of the volume osmotic flux depends on the membrane transport properties, the configuration of the membrane system as well as the physicochemical properties and composition of the solutions separated by the membrane. The common feature of these transports is that the value of this flux is higher in convective than non-convective conditions.

The purpose of the present paper was to investigate the effect of earthly gravity on concentration fields in the membrane areas. To achieve this goal, the authors will determine volume osmotic fluxes ($J_{vi}^{r})$ in a single-membrane system, in which a Nephrophan membrane (used in plate hemodialyzers) located in a horizontal plane, separates water and a ternary solution consisting of water, CuSO_4_ and/or ethanol. In addition, the authors will examine the effect of the concentration of individual solution components and the configuration of the membrane system on the value of $J_{vi}^{r}$. The study will be carried out under conditions of mechanical mixing of the solutions and after it has been turned off. Based on the obtained measurement results $J_{vi}^{r}$, the authors will calculate the effects of: concentration polarization, natural convection, asymmetry and amplification of the volume osmotic flux, as well as the thickness of concentration boundary layers. The authors will also calculate the osmotic entropy production for solution homogeneity and concentration polarization conditions as well as interpret the results obtained using the osmotic concentration polarization factor ($\zeta_{i}^{r}$). This factor, through the concentration permeability coefficient of the boundary layer ($\omega_{o}^{r})$, treated as a liquid membrane with a reflection coefficient equal to zero, will be related to the thickness of the concentration boundary layers. The thickness of these layers will be used to estimate the Rayleigh concentration number ($R_{C}^{r}$), i.e., the parameter controlling the transition from non-convective to convective state. The Rayleigh concentration number acts as a switch between two states: convective (with higher $J_{vi}^{r}$) and non-convective (with lower $J_{vi}^{r}$). The operation of this switch indicates the regulatory role of earthly gravity in relation to membrane transport.

## 2. Electrochemical Membrane Cell

**Figure 1:**
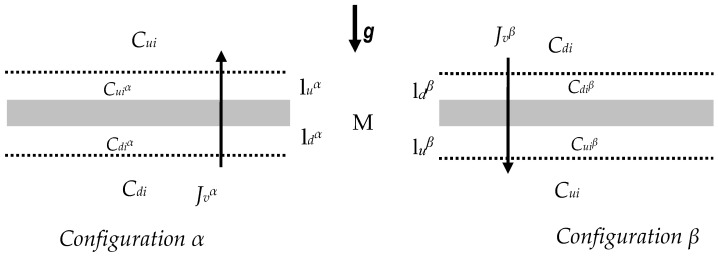
Single membrane system: M = membrane; *C_ui_* and *C_di_* (*C_ui_* > *C_di_*, *k* = 1 or 2) = solution concentrations; $J_{vi}^{\alpha }$, *J_vi_^β^* (*k* = 1 or 2) = volume flux for the *α* and *β* configuration of the membrane system, respectively.

Let us consider membrane transport in a physicochemical cell, shown in Figure 1. In this cell, the membrane (M), arranged in a horizontal plane, at the initial moment (*t*_0_ = 0), separated two homogeneous solutions of the same non-electrolytic substance with concentrations *C_ui_* i *C_di_* (*C_ui_* > *C_di_*). If the membrane in question is isotropic, symmetrical, electro-neutral and selective for water and solute, its transport properties are characterized only by the coefficients: hydraulic permeability (*L_p_*), reflection (*σ_i_*) and permeability of solute (*ω_i_*) [21]. For times satisfying the condition *t* > *t*_0_, on both sides of the membrane, the creation of concentration boundary layers begins, which change the concentration field in the areas around the membrane, generating concentration polarization [6,21].

The nature of the concentration field in the areas around the membrane is determined by the density of the solutions separated by the membrane. If the density of the solution with *C_ui_* concentration reaches a critical value in relation to the density of the solution with *C_di_* concentration, then the concentration field changes its nature from diffusive to diffusion - convective. Under the conditions of the diffusion field of concentration, the concentration of the solution, which initially was *C_ui_*, decreases to the value $C_{ui}^{\alpha d}$ or $C_{ui}^{\beta d}$, and the concentration of the solution, which initially was *C_d_*, increases to the value of $C_{di}^{\alpha d}$ or $C_{di}^{\beta d}$ ($C_{ui}^{\alpha d}$> $C_{di}^{\alpha d}$, $C_{ui}^{\beta d}$ > $C_{di}^{\beta d}$). In turn, under the conditions of diffusion-convective concentration field, the concentration of the solution that initially amounted to *C_ui_* decreases to the value $C_{ui}^{\alpha k}$ or $C_{ui}^{\beta k}$, and the concentration of the solution that initially amounted to *C_di_* increases to the value of $C_{di}^{\alpha k}$ or $C_{di}^{\beta k}$ ($C_{ui}^{\alpha k}$ > $C_{di}^{\alpha k}$, $C_{ui}^{\beta k}$ > $C_{di}^{\beta k}$). In addition, the conditions $C_{ui}^{\alpha k}$ > $C_{ui}^{\alpha d}$, $C_{di}^{\alpha k}$ > $C_{di}^{\alpha d}$, $C_{ui}^{\beta k}$ > $C_{ui}^{\beta d}$ and $C_{di}^{\beta k}$ > $C_{di}^{\beta d}$ are fullfilled.

Therefore, under the conditions of the diffusion field of concentration, on both sides of the membranes there are concentration boundary layers $l_{u}^{\alpha d}$, $l_{d}^{\alpha d}$, $l_{u}^{\beta d}$ and $l_{d}^{\beta d}$ under conditions of the diffusion-convective field of concentration; concentration boundary layers $l_{u}^{\alpha k}$, $l_{d}^{\alpha k}$, $l_{u}^{\beta k}$ and $l_{d}^{\beta k}$. The thickness of the layers $l_{u}^{\alpha k}$, $l_{d}^{\alpha k}$, $l_{u}^{\beta k}$ and $l_{d}^{\beta k}$ is much smaller than the layers $l_{u}^{\alpha d}$, $l_{d}^{\alpha d}$, $l_{u}^{\beta d}$ i $l_{d}^{\beta d}.\;$The thicknesses of layers are denoted by $\delta_{u}^{\alpha }$, $\delta_{d}^{\alpha }$, $\delta_{u}^{\beta }$ i $\delta_{d}^{\beta }$ respectively. The concentration boundary layers are treated as pseudomembranes, whose transport properties are determined by the coefficients $\sigma_{ui}^{\alpha }$ = $\sigma_{di}^{\alpha }$ = $\sigma_{ui}^{\beta }$ = $\sigma_{di}^{\beta }$ = 0 and $\omega_{ui}^{\alpha }$, $\omega_{di}^{\alpha }$, $\omega_{ui}^{\beta }$ and $\omega_{di}^{\beta }$. The volume flux through the complexes $l_{u}^{\alpha }$/M/$l_{d}^{\alpha }$ and $l_{u}^{\beta }$/M/$l_{d}^{\beta }$ will be denoted by $J_{vi}^{\alpha }$ and $J_{vi}^{\beta }$. respectively. Membrane volume transport processes occurring under the conditions of concentration polarization of areas on both sides of the membrane can be described using the first Kedem–Katchalsky equation (for volume flux) [21]. For the homogeneity conditions of diluted electrolyte solutions, this equation can be written as follows.

(1) $$J_{vi}=L_{p}\left[\left(P_{u}-P_{d}\right)\pm \overset{2}{\underset{i=1}{\sum }}\sigma_{i}f_{i}RT\left(C_{ui}-C_{di}\right)\right]$$

In turn, for concentration polarization conditions, this equation will take the form [19]

(2) $$J_{vi}^{r}=L_{p}\zeta_{p}^{r}\left[\left(P_{u}-P_{d}\right)\pm \overset{2}{\underset{i=1}{\sum }}\sigma_{i}\zeta_{i}^{r}f_{i}RT\left(C_{ui}-C_{di}\right)\right]$$

In the above equation, the coefficients of the hydrostatic permeability of the solvent and the reflection of the solute are respectively denoted by *L_p_* and *σ_i_*. In turn, $\zeta_{p}^{r}$ and $\zeta_{i}^{r}$ are the coefficients of pressure and osmotic concentration polarization, respectively. The symbol *f_i_* (1 ≤ *f_i_* ≤ 2) means the Vant Hoff coefficient. Expressions (*P_h;_ P_l_*) = Δ*P* and *RT*(*C_h_*; *C_l_*) = Δ*π* refer to the difference of respectively hydrostatic pressures (*P_h_*, *P_l_*) and osmotic pressures on both sides of the membrane (*RT* is the product of gas constant and absolute temperature and *C_h_* and *C_l_*; concentration of solutions). The coefficients $\omega_{ui}^{\alpha }$, $\omega_{di}^{\alpha }$, $\omega_{ui}^{\beta }$ and $\omega_{di}^{\beta }$ and $\delta_{u}^{\alpha }$, $\delta_{d}^{\alpha }$, $\delta_{u}^{\beta }$ and $\delta_{d}^{\beta }$ are related to the following expressions $\omega_{ui}^{\alpha }$ = $D_{ui}^{\alpha }$ (*RT*$\delta_{u}^{\alpha }$)^−1^, $\omega_{di}^{\alpha }\;$=$\;D_{di}^{\alpha }$ (*RT*$\delta_{d}^{\alpha }$)^−1^, $\omega_{ui}^{\beta }\;$= $D_{ui}^{\beta }$ (*RT*$\delta_{u}^{\beta }$)^−1^ and $\omega_{di}^{\beta }$ = $D_{di}^{\beta }$ (*RT*$\delta_{d}^{\beta }$)^−1^, where $D_{ui}^{\alpha }$, $D_{di}^{\alpha }$, $D_{ui}^{\beta }$ and $D_{di}^{\beta }$ is the appropriate diffusion coefficient. The coefficients $\zeta_{i}^{r}$, $\delta_{u}^{r}$, $\delta_{d}^{r}$, *ω_mi_*, $D_{ui}^{r}$ and $D_{di}^{r}$ are related by the equation [22]

(3) $$\zeta_{i}^{r}={\left\{1+RT\omega_{mi}\left[\frac{\delta_{u}^{r}}{{\left(D_{ui}^{r}\right)}_{l}}+\frac{\delta_{d}^{r}}{{\left(D_{di}^{r}\right)}_{h}}\right]\right\}}^{-1}$$

where: *r* = α or β and *i* = 1 or 2. This equation shows that the value of the coefficient $\zeta_{i}^{r}$ depends on the thickness of the concentration boundary layers $\delta_{u}^{r}$ i $\delta_{d}^{r}$. The process of creating these layers can be followed using a Mach-Zehnder interferometer [7,22,23]. It is also possible, based on interferograms, to determine the time-spatial evolution of the concentration field and to determine the time dependence of the concentration thicknesses of boundary layers [24]. The process of transition from diffusion to convective concentration field can be controlled by the Rayleigh concentration number (*R_C_*) [25]. Assuming that $\delta_{u}^{r}$ = $\delta_{d}^{r}$ = $\delta_{0}^{r}$, $D_{ui}^{r}$ = $D_{di}^{r}$ = $D_{i}$ this number for ternary solutions can be described by the equation [26,27]

(4) $$R_{Ci}^{r}=gRT{\left(\delta_{0}^{r}\right)}^{4}\sum_{i=1}^{2}\frac{1}{\nu_{i}D_{i}}\left\{\frac{1}{\rho_{i}}{\left(\frac{\partial \rho }{\partial C}\right)}_{i}\left[\frac{\omega_{i}\left(C_{ui}-C_{di}\right)}{2RT\omega_{i}\delta_{0}^{r}+D_{i}}\right]\right\}$$

where g is the gravitational acceleration; *ρ_i_* is the mass density, *ν_i_* is the kinematic viscosity of fluid, $\frac{1}{\rho_{i}}{\left(\frac{\partial \rho }{\partial C}\right)}_{i}$ is the variation of density with the concentration.

Entropy is produced in every membrane system, including the biological one. In the case where the driving forces in the membrane system are the differences in hydrostatic pressure (Δ*p*) and osmotic pressure (Δ*π_k_*), entropy production ($P_{S}^{r}$) can be described by the equation [10,11]

(5) $$P_{S}^{r}=T^{-1}\left[J_{vi}^{r}(\Delta p\pm \sum_{i}\Delta \pi_{i})+\sum_{i}\left(J_{i}^{r}\Delta \pi_{i}{\bar{C}}_{i}{}^{-1}\right)\right]$$

where: $J_{i}^{r}$ is the flux of *i*-th solute, ${\bar{C}}_{i}=(C_{ui}-C_{di})\left[ln(C_{ui}C_{di}{}^{-1}\right){]}^{-1}$ is the average solution concentration.

## 3. Methodology for Measuring the Volume Flux

The study of the volume osmotic flux ($J_{vi}^{r}$) was carried out using the measuring set described in the previous paper [18]. This set consisted of two cylindrical measuring vessels (U, D) made of Plexiglas with a volume of 200 cm^3^ each. Vessel U contained the tested binary or ternary solution, while vessel D had pure water. As binary solutions, aqueous CuSO_4_ solutions or aqueous ethanol solutions were used. The ternary solutions were ethanol solutions in an aqueous CuSO_4_ solution or CuSO_4_ solutions in an aqueous ethanol solution. It should be noted that the density of aqueous ethanol solutions is less than the density of water, and the density of the aqueous solution of CuSO_4_ is greater than the density of water. In turn, the density of ethanol solutions in aqueous CuSO_4_ and the density of CuSO_4_ solutions in aqueous ethanol may be less than, equal to or greater than the density of water.

The U and D vessels were separated by a cellulose acetate membrane called Nephrophan situated in a horizontal plane with an area of *S* = 3.36 cm^2^ and transport properties determined, in accordance with Kedem and Katchalsky formalism, by the factors: hydraulic permeability (*L_p_*), reflection (*σ_i_*) and diffusion permeability (*ω_i_*). The *Nephrophan* membrane is the microporous, highly hydrophilic polymeric filter used in medicine (VEB Filmfabrik, Wolfen, Germany). This membrane is made of cellulose acetate (cello-triacetate (OCO-CH_3_)_n_) [28,29]. The electron microscope image of surface and cross-section of these membrane it was presented in ref. [18]. The values of these coefficients for CuSO4 (index 1) and ethanol (index 2), determined in a series of independent experiments, are: *L_p_* = 5 × 10^−12^ m^3^N^−1^s^−1^, *σ*_1_ = 0.17, *σ*_2_ = 0.025, *ω*_1_ = 0.6 × 10^−9^ mol N^−1^s^−1^ and *ω*_2_ = 1.52 × 10^−9^ mol N^−1^s^−1^. The U vessel was connected to a graduated pipette (K) positioned in a plane parallel to the membrane plane, which was used to measure the volume increase of the solution (Δ*V*) filling the vessel. In turn, the vessel D was connected to the water reservoir (N) with adjustable height relative to the pipette K, which served to compensate for the hydrostatic pressure (Δ*p* = 0) present in the measuring set.

Each experiment was performed for the *α* and *β* configuration of the membrane system. In the *α* configuration, the test solution was in the vessel above the membrane, and the water, in the vessel under the membrane. In the *β* configuration, the order in which the solution and water were positioned relative to the membrane was reversed. The flow tests consisted of measuring the volume increase (Δ*V*) of the solution in the pipette K at 10 min intervals (Δ*t*). For each configuration, the tests were carried out according to a two-step procedure [15]. In the first stage, the volume flux was determined under mechanical mixing conditions of the solutions separated through the membrane at a speed of 500 rpm. until steady state was achieved. The second stage began with switching off the mechanical stirring of the solutions and consisted in testing the flux until the second steady state was obtained. All the investigations of volume osmotic flows were carried out under isothermal conditions for *T* = (295 ± 0.5) K. The volume osmotic flux, which is a measure of the volume osmotic flows, was calculated on the basis of the measurement of the change in volume (Δ*V*) in the pipette K occurring during Δ*t*, through the membrane surface area *S*, using the formula $J_{vi}^{r}$ = ($\Delta V_{i}^{r}$)*S*^−1^(Δ*t*)^−1^ for conditions Δ*p* = 0. The volume osmotic fluxes always occurred from the solution with a lower concentration to the solution with a higher concentration. Investigations of volume osmotic flux in both configurations consisted in determining the $J_{v1}^{\alpha }=f\left(t\right)$, $J_{v1}^{\beta }=f\left(t\right)$, $J_{v2}^{\alpha }=f\left(t\right)$ and $J_{v2}^{\beta }=f\left(t\right)$ for different concentrations and composition of solutions. Each measurement series was repeated three times. The relative error made in determining $J_{vi}^{r}$ was not greater than 3%. Based on these characteristics, for the steady state, the characteristics $J_{v1}^{\alpha }=f(\Delta C_{1},\;\Delta C_{2}$ = constant), $J_{v1}^{\beta }=f(\Delta C_{1},\;\Delta C_{2}$ = constant), $J_{v2}^{\alpha }$ = $f(\Delta C_{2},\;\Delta C_{1}$ = constant) and $J_{v2}^{\beta }=f(\Delta C_{2},\;\Delta C_{1}\;$= constant) were compiled.

## 4. Results and Discussion

**Figure 2:**
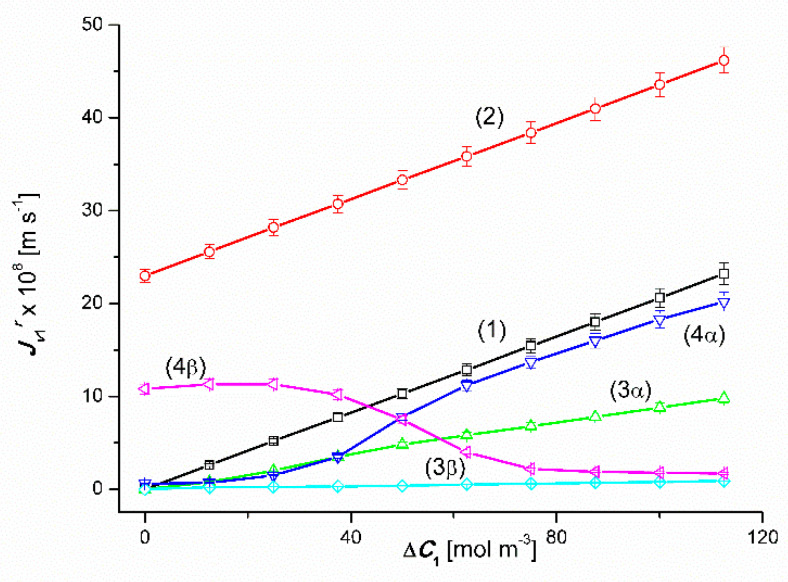
Graphic illustration of the experimental dependence $J_{v1}^{r}=f\left(\Delta C_{1},\;\Delta C_{2}=\mathrm{constant}\right)$, (*r* = α, β) for CuSO_4_ solutions in aqueous ethanol and the *α* and *β* configurations of the membrane system. Graphs 1, 3α and 3β were obtained for Δ*C*_2_ = 0, graphs 2, 4α and 4β; for Δ*C*_2_ = 750 mol m^−3^.

**Figure 3:**
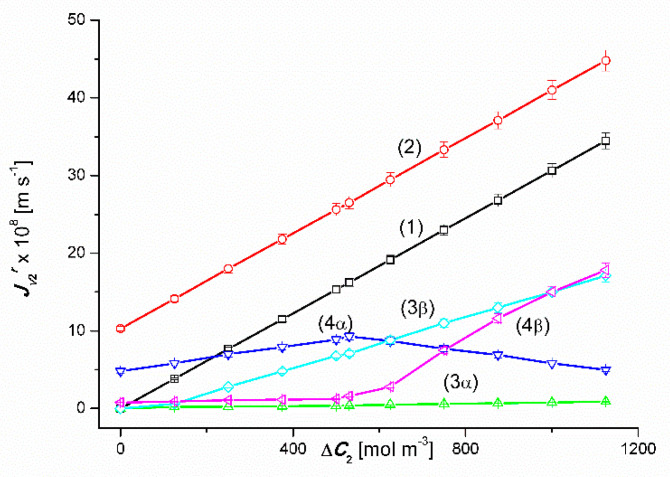
Graphic illustration of the experimental dependence $J_{v2}^{r}=f\left(\Delta C_{1},\;\Delta C_{2}=\mathrm{constant}\right)$, (*r* = α, β) for ethanol solutions in aqueous CuSO_4_ and the *α* and *β* configurations of the membrane system. Graphs 1, 3α and 3β were obtained for Δ*C*_1_ = 0, graphs 2, 4α and 4β; for Δ*C*_1_ = 50 mol m^−3^.

The results of the volume osmotic flux study for the conditions of homogeneity of solutions and conditions of concentration polarization of solutions separated by the membrane are presented in Figure 2 and Figure 3. Figure 2 shows the experimental dependences $J_{v1}^{\alpha }=f\left(\Delta C_{1},\;\Delta C_{2}=\mathrm{constant}\right)$ and $J_{v1}^{\beta }=f(\Delta C_{1},\;\Delta C_{2}$ = constant) and in Figure 3; experimental dependences $J_{v2}^{\alpha }=f(\Delta C_{2},\;\Delta C_{1}$ = constant) and $J_{v2}^{\beta }=f(\Delta C_{2},\;\Delta C_{1}$ = constant) for the *α* (*r* = α) and *β* (*r* = *β*) configurations of the membrane system, respectively. The dependences shown in Figure 1 and Figure 2 were obtained under mechanical mixing of solutions at a speed of 500 rpm. These dependences for aqueous CuSO_4_ solutions (Figure 2) and aqueous ethanol solutions (Figure 3) are linear.

Adding a fixed amount of ethanol to aqueous CuSO_4_ solutions or a fixed amount of CuSO_4_ to aqueous ethanol solutions causes a parallel shift of the line (1) by a constant and positive volume flux.

The concentration characteristics of the volume flux for concentration polarization conditions look completely different (after switching off the mechanical stirring of solutions). Graphs 3α and 3β presented in Figure 3 show that an increase in Δ*C*_1_ value in binary solutions (water solutions of CuSO_4_) for Δ*C*_2_ = 0, except for the segment 0 < Δ*C*_1_ ≤ 50 mol m^−3^, causes a linear increase in the fluxes $J_{v1}^{\alpha }$ and $J_{v1}^{\beta }$ ($J_{v1}^{\alpha }$ > $J_{v1}^{\beta }$). In turn, graphs 4α and 4β show that, unlike binary solutions, an increase in Δ*C*_1_ in ternary solutions (Δ*C*_2_ = 750 mol m^−3^), causes a non-linear increase in the value of the flux $J_{v1}^{\alpha }$ for the α configuration and an initial increase followed by a non-linear decrease in value $J_{v1}^{\beta }$ flux for the β configuration of the membrane system. In the case of the 4α curve shown in Figure 2 (Δ*C*_2_ = 750 mol m^−3^), $J_{v1}^{\alpha }$ achieves relatively small values slightly dependent on the value of Δ*C*_1_ up to Δ*C*_1_ ≤ 50 mol m^−3^. For Δ*C*_1_ > 50 mol m^−3^ $J_{v1}^{\alpha }$ reaches much higher values and strongly dependent on the value of Δ*C*_1_. The largest increase in the value of $J_{v1}^{\alpha }$ falls within the range of 37.5 mol m^−3^ < Δ*C*_1_ ≤ 62.5 mol m^−3^. In addition, for Δ*C*_1_ > 62.5 mol m^−3^), $J_{v1}^{\alpha }$ increases linearly as the Δ*C*_1_ value increases. In turn, the 4β curve shows that $J_{v1}^{\beta }$ initially decreases and for Δ*C*_1_ = 18.75 mol m^−3^ it reaches the minimum value, and then increases non-linearly with an increase in the value of Δ*C*_1_ up to Δ*C*_1_ = 100 mol m^−3^. The largest increase in the value of $J_{v1}^{\beta }$ falls within the range of 50 mol m^−3^ < Δ*C*_1_ ≥ 62.5 mol m^−3^.

Graphs 3α and 3β presented in Figure 3 show that an increase in the Δ*C*_2_ value in binary solutions (aqueous ethanol solutions) for the zero value of the concentration of CuSO_4_ (Δ*C*_1_ = 0), apart from the segment 0 < Δ*C*_2_ ≤ 200 mol m^−3^, causes a linear increase in fluxes $J_{v2}^{\alpha }$ and $J_{v2}^{\beta }$ ($J_{v2}^{\alpha }$ < $J_{v2}^{\beta }$). In turn, graphs 4*α* and 4*β* show that an increase in the value of Δ*C*_2_ in ternary solutions (Δ*C*_1_ = 50 mol m^−3^), causes a non-linear increase in the value of the flux $J_{v2}^{\alpha }$ for the α configuration and an initial increase followed by a non-linear decrease in the value of the flux $J_{v2}^{\beta }$ for the configuration *β* of membrane system. In the case of the 4*α* curve shown in Figure 3 (Δ*C*_1_ = 50 mol m^−^^3^), $J_{v2}^{\alpha }$ increases linearly until the maximum value is $J_{v2}^{\alpha }$ = 9.4 × 10^−8^ m s^−1^ for Δ*C*_2_ = 540 mol m^−3^ and then decreases linearly. It should be noted that the increments $J_{v2}^{\alpha }$ of the first segment of the 2*α* graph and the decreases in the value of the second segment of this graph are the same. In turn, the 2*β* curve shown in this figure shows that the value of $J_{v2}^{\beta }$ is initially independent of Δ*C*_2_, and then decreases non-linearly for Δ*C*_2_ ≥ 500 mol m^−3^. The largest increase in the value of $J_{v2}^{\beta }$ occurs in the range of 625 mol m^−3^ < Δ*C*_2_ ≥ 750 mol m^−3^. To sum up, the creation of concentration boundary layers, which is a consequence of turning off mechanical mixing of solutions, reduces the value of the volume osmotic flux by up to 97%. The appearance of natural convection reduces the reduction by up to 52%.

### 4.1. The Effect of Concentration Polarization

**Figure 4:**
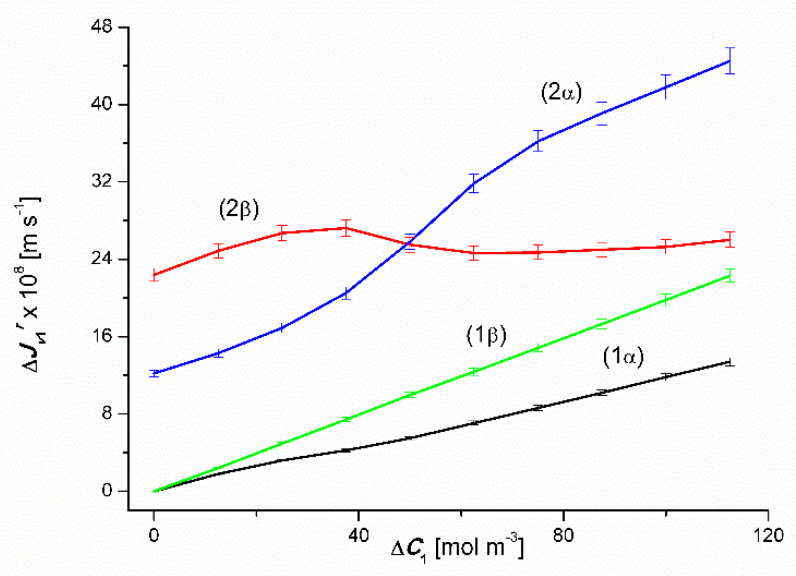
Graphic illustration of the dependence $\Delta J_{v1}^{r}=f\left(\Delta C_{1},\;\Delta C_{2}=\mathrm{constant}\right)$, (*r* = α, β) for CuSO_4_ solutions in aqueous ethanol and the *α* and *β* configurations of the membrane system. Graphs 1α and 1β were obtained for Δ*C*_2_ = 0, graphs 2α and 2β; for Δ*C*_2_ = 750 mol m^−3^.

The measure of the concentration polarization effect ($\Delta J_{vk}^{r}$) is the equation

(6) $$\Delta J_{vk}^{r}=J_{vk}-J_{vk}^{r}$$

where $J_{vk}$ is the volume osmotic flux determined for mechanical stirring conditions of solutions, $J_{vk}^{r}$ is the volume osmotic flux determined for concentration polarization conditions, *k* = 1 or 2 and *r* = *α* or *β*. Figure 4 shows the dependence $\Delta J_{v1}^{r}=f\left(\Delta C_{1},\;\Delta C_{2}=\mathrm{constant}\right)$. This graph shows that for binary solutions$\;\Delta J_{v1}^{\beta }$ > $\Delta J_{v1}^{\alpha }$ in the whole range of Δ*C*_1_. In the case of ternary solutions $\Delta J_{v1}^{\beta }$ > $\Delta J_{v1}^{\alpha }$, for Δ*C*_1_ < 47 mol m^−3^ and $\Delta J_{v1}^{\beta }$< $\Delta J_{v1}^{\alpha }$, for Δ*C*_1_ > 47 mol m^−3^.

**Figure 5:**
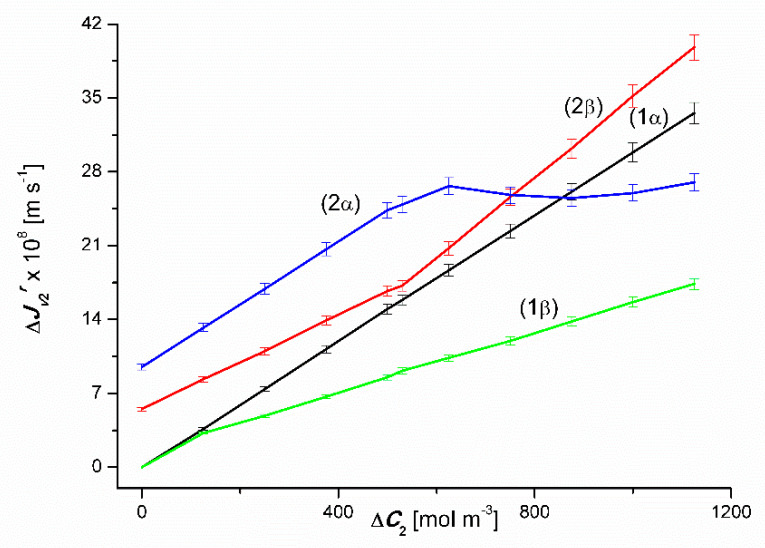
Graphic illustration of the dependence $\Delta J_{v2}^{r}=f\left(\Delta C_{2},\;\Delta C_{1}=\mathrm{constant}\right)$, (*r* = α, β) for ethanol solutions in the aqueous solution of CuSO_4_ and *α* and *β* configurations of the membrane system. Graphs 1α and 1β were obtained for Δ*C*_1_ = 0, graphs 2α and 2β; for Δ*C*_1_ = 50 mol m^−3^.

Figure 5 shows the dependencies $\Delta J_{v2}^{r}=f\left(\Delta C_{2},\;\Delta C_{1}=\mathrm{constant}\right)$. From this graph it follows that for binary solutions (Δ*C*_1_ = 0) $\Delta J_{v2}^{\beta }$ > $\Delta J_{v2}^{\alpha }$ in the whole range of Δ*C*_2_. In the case of ternary solutions (Δ*C*_1_ = 50 mol m^−3^)$\;\Delta J_{v2}^{\beta }$ < $\Delta J_{v2}^{\alpha }$ for Δ*C*_2_ < 750 mol m^−3^ and $\Delta J_{v2}^{\beta }$ > $\Delta J_{v2}^{\alpha }$, for Δ*C*_1_ > 750 mol m^−3^.

### 4.2. Convection Effect

The measure of convective effect ($\Delta J_{vk}$) is an equation

(7) $$\Delta J_{vk}=J_{vk}^{\alpha }-J_{vk}^{\beta }$$

where $J_{vk}^{\alpha }$ is the volume flux determined for concentration polarization conditions of solutions and α configuration of the membrane system, $J_{vk}^{\beta }$ is the volume flux determined for the conditions of concentration polarization of solutions and configuration of the membrane system, *k* = 1 or 2.

**Figure 6:**
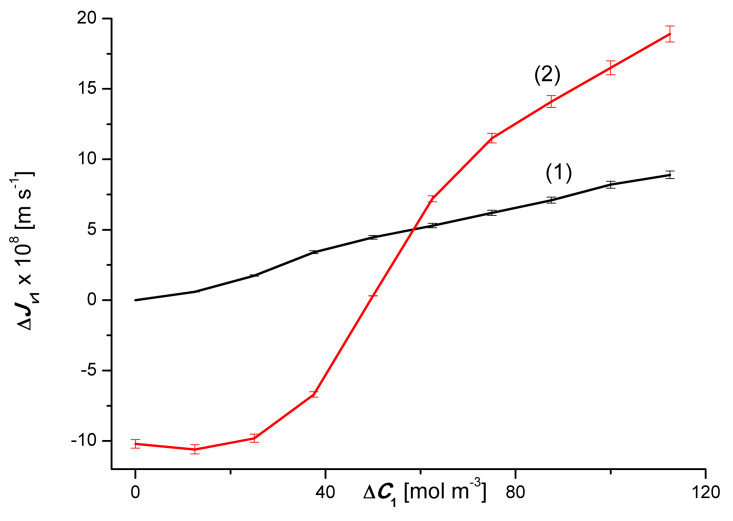
Graphic illustration of the dependence $\Delta J_{v1}=f\left(\Delta C_{1},\;\Delta C_{2}=\mathrm{constant}\right)$, (*r* = α, β) for CuSO_4_ solutions in aqueous ethanol and the *α* and *β* configurations of the membrane system. Graph 1 was obtained for Δ*C*_2_ = 0, graph 2; Δ*C*_2_ = 750 mol m^−3^.

Figure 6 shows the dependence $\Delta J_{v1}=f\left(\Delta C_{1},\;\Delta C_{2}=\mathrm{constant}\right)$. This graph shows that for binary solutions (Δ*C*_2_ = 0) $\Delta J_{vk}$ > 0 in the whole range of Δ*C*_1_. For ternary solutions (Δ*C*_2_ = 750 mol m^−3^), $\Delta J_{vk}$ < 0 for Δ*C*_1_ < 47 mol m^−3^ and $\Delta J_{vk}$ > 0, for Δ*C*_1_ > 47 mol m^−3^.

**Figure 7:**
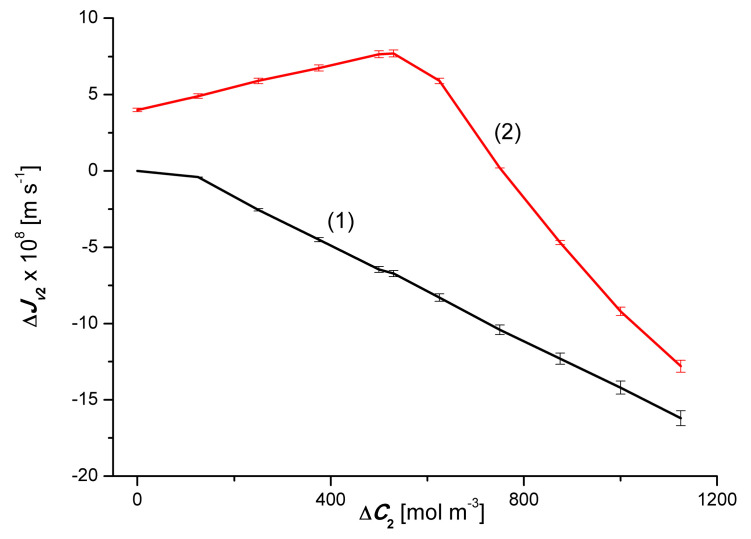
Graphic illustration of the dependence $\Delta J_{v2}=f\left(\Delta C_{2},\;\Delta C_{1}=\mathrm{constant}\right)$, (*r* = α, β) for ethanol solutions in aqueous CuSO_4_ solution and *α* and *β* configurations of the membrane system. Graph 1 was obtained for Δ*C*_1_ = 0, graph 2; for Δ*C*_1_ = 50 mol m^−3^.

Figure 7 shows the dependence $\Delta J_{v2}=f\left(\Delta C_{2},\;\Delta C_{1}=\mathrm{constant}\right)$. This graph shows that for binary solutions (Δ*C*_1_ = 0), $\Delta J_{v2}$ < 0 in the whole range of Δ*C*_2_. For ternary solutions (Δ*C*_1_ = 50 mol m^−3^),$\;\Delta J_{v2}$ > 0, for Δ*C*_2_ < 750 mol m^−3^, and $\Delta J_{v2}$ < 0, for Δ*C*_2_ > 750 mol m^−3^. It should be noted that the test results presented in Figure 6 and Figure 7 are similar to the results of studies on the gravity-osmotic flux measured in a two-membrane system [14,15]. The membranes in this system were horizontally oriented and separated aqueous solutions of glucose and/or ethanol. The concentrations of these solutions met the condition *C_ui_* = *C_di_* < *C_mi_* (*C_ui_*, *C_di_*; solution concentrations in the external compartments, *C_mi_*; solution concentration in the inter-membrane compartment). The equivalent of such a membrane system is two single-membrane systems connected in parallel.

### 4.3. The Effect of Asymmetry of the Volume Osmotic Flux

**Figure 8:**
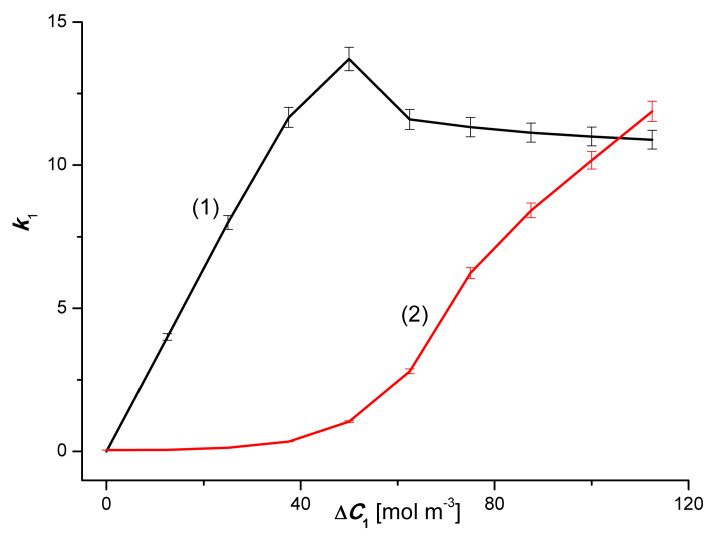
Graphic illustration of the dependence *k*_1_ = *f*(Δ*C*_1_, Δ*C*_2_ = constant). For solutions of CuSO_4_ in aqueous ethanol. Graphs 1 and 2 were obtained for Δ*C*_2_ = 0 and Δ*C*_2_ = 750 mol m^−3^, respectively.

**Figure 9:**
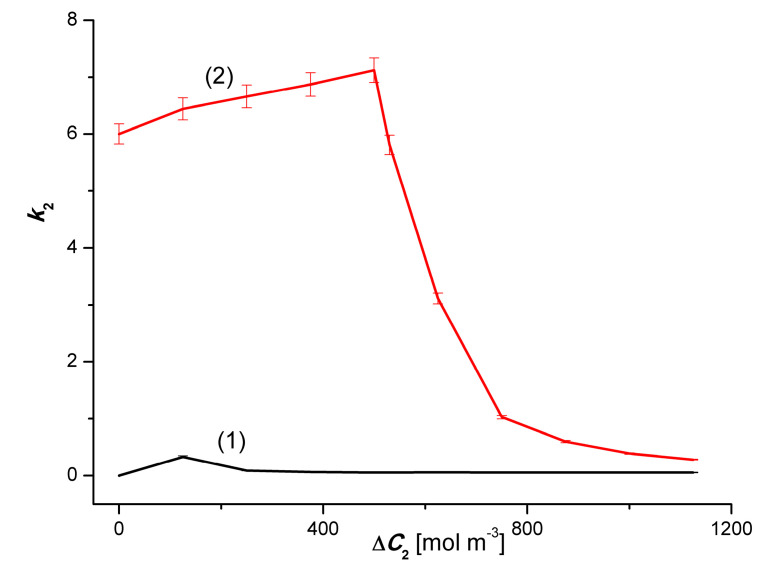
Graphic illustration of the dependence *k*_2_ = *f*(Δ*C*_2_, Δ*C*_1_ = constant) for ethanol solutions in aqueous CuSO_4_. Graphs 1 and 2 were obtained for Δ*C*_1_ = 0 and Δ*C*_1_ = 50 mol m^−3^, respectively.

The comparison of the 3α and 3β and 4α and 4β plots presented in Figure 2 and Figure 3 shows the asymmetry of the volume osmotic fluxes, which is the evidence of the osmotic rectifying properties of the membrane system. The measure of this asymmetry is the asymmetry coefficients *k*_1_= $J_{v1}^{\alpha }$/$J_{v1}^{\beta }$ and *k*_2_ = $J_{v2}^{\alpha }$/$J_{v2}^{\beta }$. The curves in Figure 8 and Figure 9 show the characteristics of *k*_1_ = *f*(Δ*C*_1_, Δ*C*_2_ = constant) and *k*_2_ = *f*(Δ*C*_2_, Δ*C*_1_ = constant). Graphs 1 in Figure 8 and Figure 9 illustrate the dependences *k*_1_ = *f*(Δ*C*_1_, Δ*C*_2_ = 0) and *k*_2_ = *f*(Δ*C*_2_, Δ*C*_1_ = 0). respectively. In turn, graphs 2 presented in these graphs illustrate the *k*_1_ = *f*(Δ*C*_1_, Δ*C*_2_ = 750 mol m^−3^) and *k*_2_ = *f*(Δ*C*_2_, Δ*C*_1_ = 50 mol m^−3^). The values of *k*_1_ and *k*_2_ coefficients, different from unity, indicate that the tested membrane system has rectifying properties, which are manifested as the asymmetry of the volume osmotic flux.

### 4.4. The Effect of Amplification the Volume Osmotic Flux

The measure of the amplification effect of the osmotic volume flux is the amplification coefficient, the definition of which is the equation

(8) $$a_{vk}^{r}=\frac{{\left(\Delta J_{vk}^{r}\right)}_{ternary}}{{\left(\Delta J_{vk}^{r}\right)}_{binary}}$$

where ${\left(\Delta J_{vk}^{r}\right)}_{ternary}$ is the volume flux increase for ternary solutions, ${\left(\Delta J_{vk}^{r}\right)}_{binary}$ is the volume flux increase for ternary solutions, *k* = 1 or 2 and *r* = *α* or *β*.

**Figure 10:**
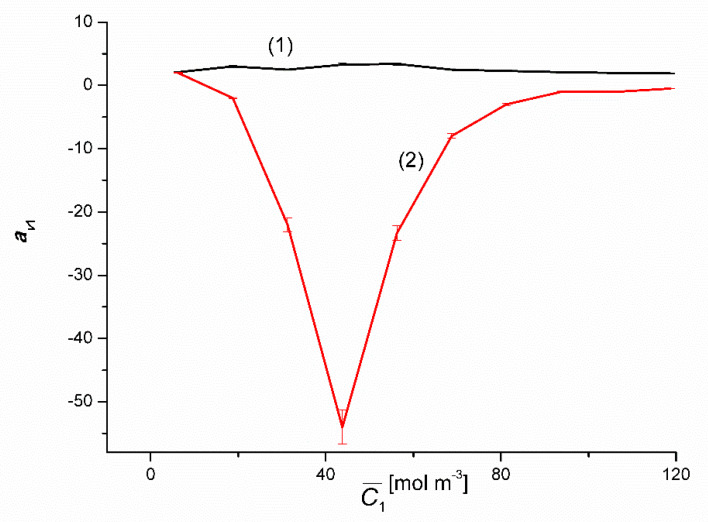
Graphic illustration of the dependence $a_{v1}^{r}\;$= *f*(${\bar{C}}_{1}$, Δ*C*_2_ = constant) for solutions of CuSO_4_ in aqueous ethanol. Graphs 1 and 2 were obtained for Δ*C*_2_ = 0 and Δ*C*_2_ = 750 mol m^−3^, respectively.

**Figure 11:**
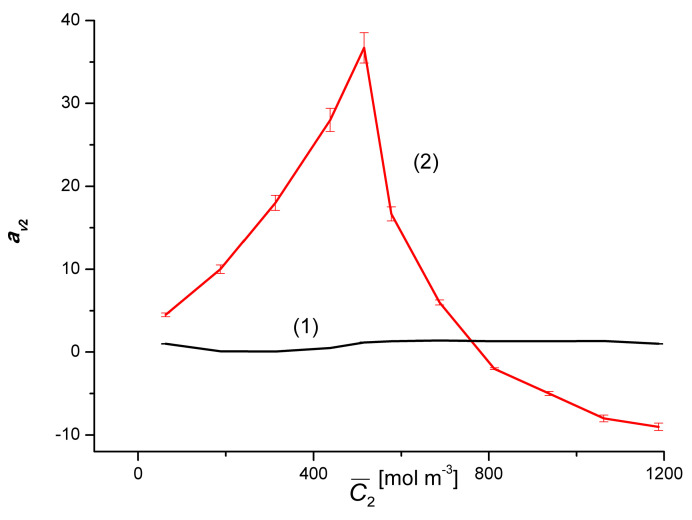
Graphic illustration of the relationship $a_{v2\;}^{r}$= *f*(${\bar{C}}_{2}$, Δ*C*_1_ = constant) for solutions of ethanol in an aqueous solution of CuSO_4_. Graphs 1 and 2 were obtained for Δ*C*_1_ = 0 and Δ*C*_1_ = 50 mol m^−3^, respectively.

Figure 10 and Figure 11 show the dependencies $a_{vk}^{r}=f\left({\bar{C}}_{1},\;\Delta C_{2}=\mathrm{constant}\right)$, where ${\bar{C}}_{1}$ = 0.5(C_j_ + C_j__+1_), j = 1, 2, …). Figure 10 shows that for binary solutions (Δ*C*_2_ = 0) $a_{v1}^{r}$ > 0 in the whole range ${\bar{C}}_{1}$ and takes values from $a_{v1}^{r}$ = 2.1 to $a_{v1}^{r}$ = 3.3. In the case of ternary solutions (Δ*C*_2_ = 750 mol m^−3^), the dependence $a_{vk}^{r}=f\left({\bar{C}}_{1},\;\Delta C_{2}=\mathrm{constant}\right)$ is nonlinear, with a clearly marked minimum, and the coefficient $a_{v1}^{r}$ is negative. The minimum of this dependence has the coordinates ${\bar{C}}_{1}$ = 43.75 mol m^−3^ and $a_{v1}^{r}$ = −54.

In turn, Figure 11 shows that for binary solutions (Δ*C*_1_ = 0), $a_{v2}^{r}$ > 0 in the whole range ${\bar{C}}_{2}$ and takes values from $a_{v2}^{r}$ = 0.5 to $a_{v2}^{r}$ = 1.4. In the case of ternary solutions (Δ*C*_1_ = 50 mol m^−3^), the dependence $a_{v2}^{r}=f\left({\bar{C}}_{2},\;\Delta C_{1}=\mathrm{constant}\right)$ is non-linear, with the maximum clearly indicated, and the coefficient $a_{v2}^{r}$ assumes positive values for ${\bar{C}}_{2}$ < 760 mol m^−3^ and negative for ${\bar{C}}_{2}$ < 760 mol m^−3^. The maximum of this dependence has the coordinates ${\bar{C}}_{2}$ = 515.75 mol m^−3^ and $a_{v2}^{r}$ = 36.7. Rectifying properties along with amplification properties and oscillation generation belong to the group of regulatory phenomena [19].

### 4.5. Evaluation of Osmotic Entropy Production

The osmotic entropy production ($P_{S}^{r}$) will be calculated using Equation (5), omitting the term $\underset{i}{\sum }\left(J_{i}^{r}\Delta \pi_{i}{\bar{C}}_{i}{}^{-1}\right)$ and assuming that Δ*p* = 0 and *i* = 1, 2. With such assumptions the Equation (5) will take the form

(9) $$P_{S}^{r}=J_{vi}^{r}R\left[\left(C_{u1}-C_{d1}\right)+\left(C_{u2}-C_{d2}\right)\right]$$

**Figure 12:**
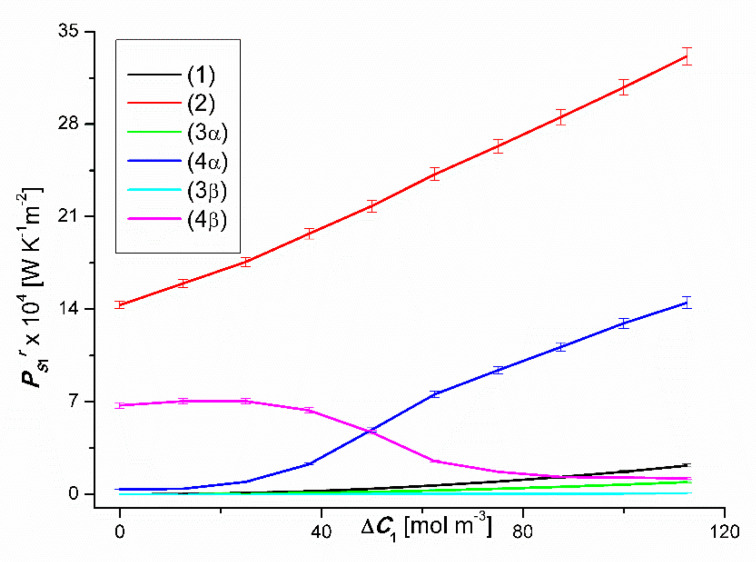
Graphic illustration of the dependencies $P_{S1}^{r}=f\left(\Delta C_{1},\;\Delta C_{2}=\mathrm{constant}\right)$, (*r* = α, β) for CuSO_4_ solutions in aqueous ethanol and the *α* and *β* configurations of the membrane system. Graphs 1, 3α and 3β were obtained for Δ*C*_2_ = 0, graphs 2, 4α and 4β; for Δ*C*_2_ = 750 mol m^−3^.

**Figure 13:**
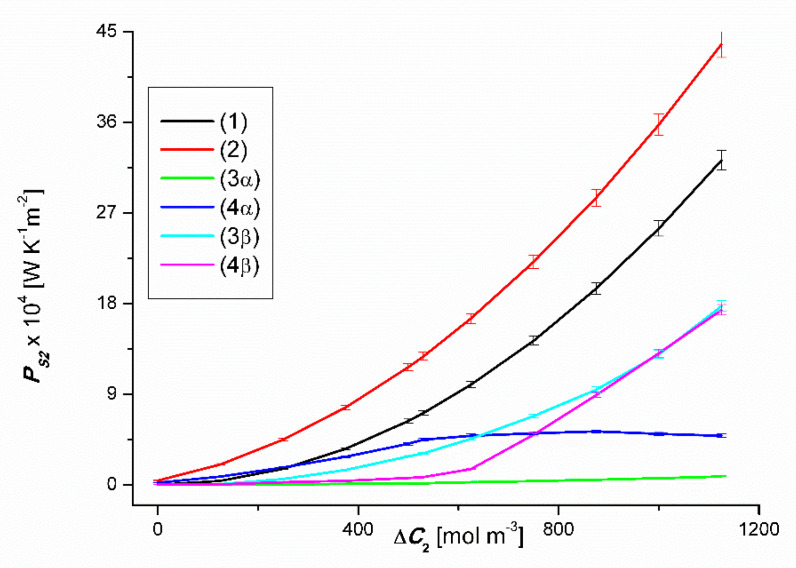
Graphic illustration of the dependencies $P_{S2}^{r}=f\left(\Delta C_{1},\;\Delta C_{2}=\mathrm{constant}\right)$, (*r* = α, β) for ethanol solutions in aqueous CuSO_4_ and the *α* and *β* configurations of the membrane system. Graphs 1, 3α and 3β were obtained for Δ*C*_1_ = 0, graphs 2, 4α i 4β; for Δ*C*_1_ = 50 mol m^−3^.

This equation shows that $P_{S}^{r}$ is directly proportional to, among others, $J_{vi}^{r}$. Taking into account the results of $J_{vi}^{r}$ presented in Figure 2 and Figure 3 in the above equation, the relationships $P_{S1}^{r}=f\left(\Delta C_{1},\;\Delta C_{2}=\mathrm{constant}\right)$ and $P_{S2}^{r}=f\left(\Delta C_{1},\;\Delta C_{2}=\mathrm{constant}\right)$, (*r* = α, β). The results of the calculations are presented in Figure 12 and Figure 13. These figures show that for the same values $\Delta C_{1}$ i $\Delta C_{2}$, both $P_{S1}^{r}$ and $P_{S2}^{r}$ follow the changes in the values of $J_{v1}^{r}$ or $J_{v2}^{r}$. Under the conditions of homogeneity of the solutions $P_{S1}^{r}$ and $P_{S2}^{r}$ they increase with the increase of the values of $J_{v1}^{r}$ and $J_{v2}^{r}$, respectively. On the other hand, under the conditions of concentration polarization, the values $P_{S1}^{r}$ and $P_{S2}^{r}$ increase when free convection appears in the membrane system and decreases when convection disappears. Due to the fact that concentration polarization reduces $J_{v1}^{r}$ and $J_{v2}^{r}$, it also reduces $P_{S1}^{r}$ and $P_{S2}^{r}$.

Equations (2)–(4) will be used to interpret the results of osmotic volume flux tests for concentration polarization conditions and presented in Figure 2 and Figure 3. For this purpose, Equation (2), for *p_u_*; *p_d_* = 0, will be transformed into the form

(10) $$\zeta_{i}^{r}=\frac{J_{vi}^{r}}{RTL_{p}\left[\sigma_{1}f_{1}\left(C_{u1}-C_{d1}\right)+\sigma_{2}f_{2}\left(C_{u2}-C_{d2}\right)\right]}$$

Having Equation (3) in the above equation, we get

(11) $$\frac{1}{1+RT\omega_{mi}\left(\frac{\delta_{u}^{r}}{D_{ui}^{r}}+\frac{\delta_{d}^{r}}{D_{di}^{r}}\right)}=\frac{J_{vi}^{r}}{RTL_{p}\sum_{i=1}^{2}\sigma_{i}f_{i}\left(C_{ui}-C_{di}\right)}$$

Assuming that $\delta_{u}^{r}$ = $\delta_{u}^{r}$ = $\delta_{i}^{r}$, $D_{ui}^{r}$ = $D_{di}^{r}$ = $D_{i}$ and *f*_2_ = 1, the equation can be written in a simplified form, namely

(12) $$\delta_{i}^{r}=\frac{D_{i}}{2RT\omega_{mi}}\left[\frac{RTL_{p}\sum_{i=1}^{2}\sigma_{i}f_{i}\left(C_{ui}-C_{di}\right)}{J_{vi}^{r}}-1\right]$$

**Figure 14:**
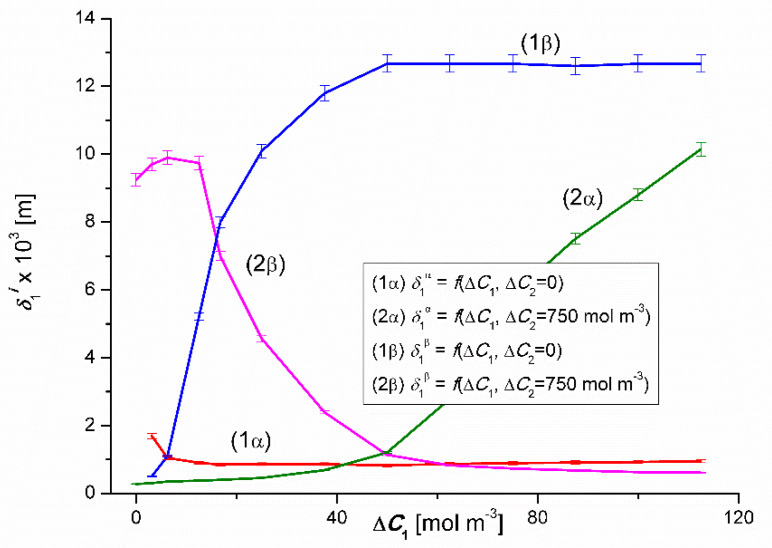
Graphic illustration of the dependencies $\delta_{i}^{r}=f\left(\Delta C_{1},\;\Delta C_{2}=\mathrm{constant}\right)$, (*r* = α, β; *i* = 1, 2) for CuSO_4_ solutions in aqueous ethanol solution and *α* and *β* configurations of membrane system. Graphs 1α and 1β were obtained for Δ*C*_2_ = 0, graphs 2α and 2β; for Δ*C*_2_ = 750 mol m^−3^.

**Figure 15:**
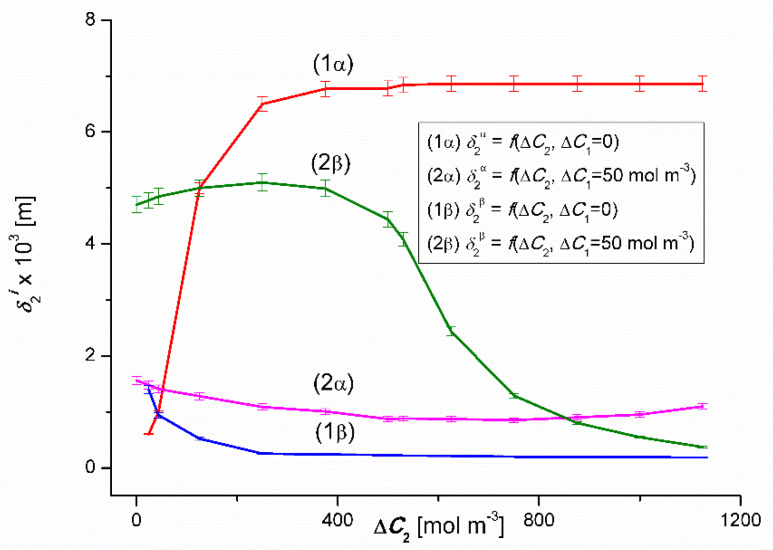
Graphic illustration of the dependencies $\delta_{2}^{r}=f\left(\Delta C_{1},\;\Delta C_{2}=\mathrm{constant}\right)$ (*r* = α, β) for ethanol solutions in aqueous CuSO_4_ solution and *α* and *β* configurations of the membrane system. Graphs 1α and 1β were obtained for Δ*C*_1_ = 0, graphs 2α and 2β; for Δ*C*_1_ = 50 mol m^−3^.

Based on Equation (10), the dependencies $\delta_{1}^{\alpha }=f(\Delta C_{1},\;\Delta C_{2}$ = constant), $\delta_{1}^{\beta }=f(\Delta C_{1},\;\Delta C_{2}$ = constant), $\delta_{2}^{\alpha }=f(\Delta C_{2},\;\Delta C_{1}$ = 50 mol m^−3^) and $\delta_{2}^{\beta }=f(\Delta C_{2},\;\Delta C_{1}$ = 50 mol m^−3^) were calculated. The following data was used for *R_C_* calculations: *D*_1_ = 0.73 × 10^−9^ m^2^s^−1^, *D*_2_ = 1.37 × 10^−9^ m^2^s^−1^, *R* = 8.31 J mol^−1^K^−1^, *T* = 295 K, *L_p_* = 5 × 10^−12^ m^3^N^−1^s^−1^, *σ*_1_ = 0.17, *σ*_2_ = 0.025, *ω_m_*_1_ = 0.6 × 10^−9^ mol N^−1^s^−1^ and *ω_m_*_2_ = 1.52 × 10^−9^ mol N^−1^s^−1^, *f*_1_ = 2 and *f*_2_ = 1. The results of the calculations are illustrated in Figure 14 and Figure 15. 

The curves 1α and 1β presented in Figure 14 illustrate the dependencies $\delta_{1}^{\alpha }=f(\Delta C_{1},\;\Delta C_{2}$ = 0) and $\delta_{1}^{\beta }=f(\Delta C_{1},\;\Delta C_{2}$ = 0), while the curves 2α and 2β – dependencies $\delta_{1}^{\alpha }=f(\Delta C_{1},\;\Delta C_{2}$ = 750 mol m^−3^) and $\delta_{1}^{\beta }=f(\Delta C_{1},\;\Delta C_{2}$ = 750 mol m^−3^). From the course of the 1α and 1β curves, it can be seen that the values of $\delta_{1}^{\alpha }$ decrease and $\delta_{1}^{\beta }$ – increase non-linearly. For Δ*C*_1_ = 5.1 mol m^−3^ $\delta_{1}^{\alpha }$ = $\delta_{1}^{\beta }$= 1.02 × 10^−3^ m, which means that the value of $\delta_{1}^{r}$ is independent of the configuration of the membrane system and thus also of the dependence between the gravity vector and the density gradient of binary solutions separated through the membrane. For Δ*C*_1_ ≥ 25 mol m^−3^, the value of $\delta_{1}^{\alpha }$ is approximately constant and amounts to about $\delta_{1}^{\alpha }$ = 0.9 × 10^−3^ m and for Δ*C*_1_ ≥ 50 mol m^−3^ $\delta_{1}^{\beta }$ = 12.7 × 10^−3^ m = constant, and therefore $\delta_{1}^{\alpha }$ < $\delta_{1}^{\beta }$. This means that for Δ*C*_1_ ≥ 25 mol m^−3^ and the α configuration of the membrane system, convection fluxes generated in the membrane areas destroy the concentration boundary layers, increasing the volume flux through the membrane.

For the 2α and 2β curves in this figure, the values of $\delta_{1}^{\alpha }$ initially increase linearly and then, after reaching the maximum value $\delta_{1}^{\alpha }$ = 9.9 × 10^−3^ m for Δ*C*_1_ = 6.25 mol m^−3^ decrease non-linearly. In turn, the values of $\delta_{1}^{\beta }$ increase non-linearly. For Δ*C*_1_ = 50 mol m^−3^ $\delta_{1}^{\alpha }$ = $\delta_{1}^{\beta }$= 1.02 × 10^−3^ m, which means that the value of $\delta_{1}^{r}$ is independent of the configuration of the membrane system and thus also of the dependence between the gravity vector and the density gradient of ternary solutions separated through the membrane. Comparing graphs 2α and 2β, it can be seen that for Δ*C*_1_ < 50 mol m^−3^, $\delta_{1}^{\alpha }$ < $\delta_{1}^{\beta }$ while for Δ*C*_1_ > 50 mol m^−3^, $\delta_{1}^{\alpha }$ > $\delta_{1}^{\beta }$. This means that for Δ*C*_1_ > 50 mol m^−3^ and the β configuration of the membrane system (curve 2β), and for Δ*C*_1_ < 50 mol m^−3^ and the configuration of the membrane system (curve 2α), the convection fluxes generated in the membrane areas cause concentration destruction of boundary layers, increasing the volume flow through the membrane.

The curves 1α and 1β presented in Figure 15 illustrate the dependencies $\delta_{2}^{\alpha }=f(\Delta C_{2},\;\Delta C_{1}$ =0) and $\delta_{2}^{\beta }=f(\Delta C_{1},\;\Delta C_{2}$ = 0), while the curves 2α and 2β – dependencies $\delta_{2}^{\alpha }=f(\Delta C_{2},\;\Delta C_{1}=$ 50 mol m^−3^) and $\delta_{2}^{\beta }=f(\Delta C_{2},\;\Delta C_{1}$ = 50 mol m^−3^). From the course of the 1α and 1β curves, it can be seen that the values of $\delta_{2}^{\alpha }$ initially increase non-linearly and $\delta_{2}^{\beta }$ – decrease non-linearly. For Δ*C*_2_ = 50 mol m^−3^, $\delta_{2}^{\alpha }$ = $\delta_{2}^{\beta }$ = 0.94 × 10^−3^ m, which means that the value of $\delta_{2}^{r}$ is independent of the configuration of the membrane system and thus also of the dependence between the gravity vector and the density gradient of binary solutions separated through the membrane. For Δ*C*_2_ ≥ 375 mol m^−3^ $\delta_{2}^{\alpha }$ = 6.8 × 10^−3^ m = const. and for Δ*C*_2_ ≥ 375 mol m^−3^ $\delta_{2}^{\beta }$ = 0.2 × 10^−3^ m = const., and therefore $\delta_{2}^{\alpha }$ > $\delta_{2}^{\beta }$. This means that for Δ*C*_2_ ≥ 375 mol m^−3^ in the β configuration of the membrane system, convection fluxes generated in the membrane regions destroy the concentration boundary layers, increasing the volume flow through the membrane.

In the case of the 2α and 2β curves in this figure, the values of $\delta_{2}^{\beta }$ initially increase and then, after reaching the maximum value $\delta_{2}^{\beta }$ = 5.1 × 10^−3^ m for Δ*C*_2_ = 250 mol m^−3^ decrease non-linearly. In turn, the values of $\delta_{2}^{\alpha }$ change non-linearly. For Δ*C*_2_ = 850 mol m^−3^ $\delta_{2}^{\alpha }$ = $\delta_{2}^{\beta }$ = 0.92 ×10^−3^ m, which means that the value of $\delta_{2}^{r}$ is independent of the configuration of the membrane system and thus also of the dependence between the gravity vector and the density gradient of ternary solutions separated through the membrane. Comparing graphs 2α and 2β, it can be seen that for Δ*C*_2_ < 840 mol m^−3^ $\delta_{2}^{\alpha }$ < $\delta_{2}^{\beta }$, while for Δ*C*_2_ > 840 mol m^−3^, $\delta_{2}^{\alpha }$ > $\delta_{2}^{\beta }$. This means that for Δ*C*_1_ > 840 mol m^−3^ and the β configuration of the membrane system (graph 2β), and for Δ*C*_1_ < 840 mol m^−3^ and the α configuration of the membrane system (graph 2 α), the convection fluxes generated in the membrane areas cause concentration destruction of the boundary layers, increasing the volume flux through the membrane.

As already mentioned, the Rayleigh concentration number ($R_{C}^{r}$), which is the parameter controlling the transition from non-convective to convective state can be expressed using Equation (4). We assume that at the point where $\delta_{i}^{\alpha }$ = $\delta_{i}^{\beta }$ = ${\left(\delta_{i}\right)}_{crit.}$, (*i* = 1, 2) the concentration number meets the condition $R_{Ci}^{\alpha }$ = $R_{Ci}^{\beta }$ = ${\left(R_{Ci}\right)}_{crit.}$. Calculations ${\left(R_{Ci}\right)}_{crit.}$ will be made for the following data *D*_1_ = 0.73 × 10^−9^ m^2^s^−1^, *D*_2_ = 1.37 × 10^−9^ m^2^s^−1^, *ω*_1_ = 0.6 × 10^−9^ mol N^−1^s^−1^, *ω*_2_ = 1.52 × 10^−9^ mol N^−1^s^−1^, *ρ*_0_ = 998 kg m^−3^, *ν*_0_ = 1.012 × 10^−6^ m^2^s^−1^, (*∂ρ/∂C*_1_)_bin._ = 0.06 kg mol^−1^ (for Δ*C*_2_ = 0), (*∂ρ/∂C*_1_)_ter._ = 0.05 kg mol^−1^ (for Δ*C*_2_ = 750 mol m^−3^), (*∂ρ/∂C*_2_)_bin._ = −0.0095 kg mol^−1^ (for Δ*C*_1_ = 0) and (*∂ρ/∂C*_2_)_ter._ = −0.0035 kg mol^−1^ (for Δ*C*_1_ = 50 mol m^−3^). It should be noted that *∂ρ/∂C*_i_ (*i* = 1, 2) is added for solutions whose density increases with increasing concentration and negative – when the density of solutions decreases with increasing concentration. Therefore, the indication ${\left(R_{Ci}\right)}_{crit.}$ is determined by the indication ∂ρ/∂C_i_. For calculations the values of $\delta_{i}^{\alpha }$ = $\delta_{i}^{\beta }$ = ${\left(\delta_{i}\right)}_{crit.}$ (*i* = 1, 2) will be used, taken from the curves presented in Figure 14 and Figure 15. Graphs 1α and 1β intersect at a point with coordinates Δ*C*_1_ = 5.1 mol m^−3^ and $\delta_{1}^{\alpha }$ = $\delta_{1}^{\beta }$= 1.02 × 10^−3^ m. Taking the relevant data into Equation (4) gives ${\left(R_{C1}\right)}_{crit.}$= 1737.89. In turn, the curves 2α and 2β presented in Figure 14 intersect at a point with coordinates Δ*C*_1_ = 50 mol m^−3^ and $\delta_{1}^{\alpha }$ = $\delta_{1}^{\beta }$= 1.02 × 10^−3^ m. Therefore, taking into account relevant data in Equation (4) gives ${\left(R_{C2}\right)}_{crit.}$ = 1335.69. Figure 15 shows that the diagrams 1α and 1β intersect at a point with the coordinates Δ*C*_2_ = 50 mol m^−3^ $\delta_{2}^{\alpha }$ = $\delta_{2}^{\beta }$ = 0.92 × 10^−3^ m. Therefore, taking into account the relevant data in Equation (4) gives ${\left(R_{C2}\right)}_{crit.}$ = −1169.79. Figure 15 also shows that the graphs 2α and 2β intersect at a point with the coordinates Δ*C*_2_ = 850 mol m^−3^ $\delta_{2}^{\alpha }$ = $\delta_{2}^{\beta }$ = 0.92 ×10^−3^ m. Therefore, taking into account relevant data in Equation (4) we get ${\left(R_{C2}\right)}_{crit.}$ = −1408.68.

Graphs 1α and 1β show that for $R_{C1}$ <$\;{\left(R_{C1}\right)}_{kryt.}$ and $R_{C2}$ >$\;{\left(R_{C2}\right)}_{kryt.}$ non-convective state in both configurations of the membrane system is being dealt with. $R_{C1}$ >$\;{\left(R_{C1}\right)}_{kryt.}$ in the α configuration (graphs 1α and 2α) a convective state is obtained and in the β configuration (graphs 1β and 2β) – the non-convective state. On the other hand, for $R_{C2}$ <$\;{\left(R_{C2}\right)}_{kryt.}$ in the α configuration (graphs 1α and 2α) a non-convective state is obtained, and for the β configuration (graphs 1β and 2β); the convective state. Therefore, the authors have shown that the concentration Rayleigh number ($R_{C}^{r}$) is a parameter controlling the transition from non-convective to convective state. This number also acts as a switch between two convective states (with a higher $J_{vi}^{r}$ value) and non-convective states (with a lower $J_{vi}^{r}$ value). The operation of this switch indicates the regulatory role of earthly gravity in relation to membrane transport.

Investigations on membrane transport are one of the most forward-looking directions in biotechnology, biomedical engineering and environmental protection and engineering, especially in water treatment and purification. Moreover, in recent years the research on integrated membrane processes has also been carried out [30]. The research results presented in the paper may also be relevant for nature-inspired chemical engineering (NICE) [31].

## 5. Conclusions

In this article, the authors presented the results of studies on the impact of the concentration of individual solution components and the configuration of the membrane system on the value of the volume osmotic flux $\left(J_{vi}^{r}\right)$ in a single-membrane system, in which the polymer membrane was positioned in a horizontal plane and separated water and a ternary solution consisting of water, ethanol and/or CuSO_4_. From the studies it results, that for conditions of concentration polarization and binary solutions $J_{vi}^{r}$ is a linear and for ternary solutions a non-linear function of the solution concentration differences. In addition, $J_{vi}^{r}$ depends on the configuration of the membrane system. For mechanically stirred solutions, $J_{vi}^{r}$ is independent of the membrane system configuration and is a linear function of the difference in solution concentrations. The effects of concentration polarization, convective polarization, asymmetry and amplification of the volume osmotic flux calculated on the basis of $J_{vi}^{r}$ measurements are a consequence of the concentration polarization of solutions adjacent to the membrane. The effects of concentration polarization and convective polarization for binary solutions are linear and for ternary ones a non-linear function of the concentration difference. The measures of asymmetry and amplification of the volume osmotic flux (which are a consequence of concentration polarization) are the corresponding asymmetry coefficients *k*_1_ and *k*_2_ and the amplification coefficients *a_v_*_1_ and *a_v_*_2_. The *k*_1_ coefficient for both binary and ternary solutions is a non-linear function of the difference in concentration of CuSO_4_. In turn, the value of the coefficient *k*_2_ for binary solutions is independent of the concentration and for ternary solutions; it is a non-linear function of the difference in ethanol concentration. For binary solutions, the values of *a_v_*_1_ and *a_v_*_2_ coefficients are constant and positive. In turn, for ternary solutions, these coefficients are a non-linear function of the respective concentration differences and assume both positive and negative values.

It has been shown that entropy production occurs in the single-membrane system study, which is a consequence of two thermodynamic forces (one variable and the other constant) and the generation of an osmotic flux. It has been shown, that the factor $\zeta_{i}^{r}$, by the thickness of the concentration boundary layer ($\delta_{i}^{r})$, can be associated with the Rayleigh concentration number ($R_{C}^{r}$), i.e., the parameter controlling the transition from non-convection (diffusion) to convective concentration field. Four different concentration Rayleigh number, which differ in values and signs were obtained.

The $R_{C}^{r}$ signs is conditioned by the relationship between the gravity vector and the solution density gradient. It has been shown that this number also acts as a switch between two states of the concentration field: convective (with a higher $J_{vi}^{r}$ value) and non-convective (with a lower $J_{vi}^{r}$ value). The operation of this switch indicates the regulatory role of earthly gravity in relation to membrane transport.
